# Profound influence of different methods for determination of the ankle brachial index on the prevalence estimate of peripheral arterial disease

**DOI:** 10.1186/1471-2458-7-147

**Published:** 2007-07-06

**Authors:** Stefan F Lange, Hans-Joachim Trampisch, David Pittrow, Harald Darius, Matthias Mahn, Jens R Allenberg, Gerhart Tepohl, Roman L Haberl, Curt Diehm, the getABI Study Group

**Affiliations:** 1 Department of Medical Informatics, Biometry and Epidemiology, University of Bochum, Universitätsstr., 150D-44801 Bochum, Germany; 2 Department of Clinical Pharmacology, Medical Faculty, Technical University of Dresden, Dresden, Germany; 3 Department of Medicine I, Vivantes Berlin-Neukölln Medical Centre, Berlin, Germany; 4 Medical Department, Sanofi-Aventis, Geneva, Switzerland; 5 Department of Vascular Surgery, Ruprecht-Karls University, Heidelberg, Germany; 6 Internist/Vascular Medicine, Munich, Germany; 7 Department of Neurology, Municipal Hospital Munich-Harlaching, Germany; 8 Department of Internal Medicine/Vascular Medicine, Klinikum Karlsbad-Langensteinbach, Affiliated Teaching Hospital of the Ruprecht-Karls University of Heidelberg, Germany; 9 Institut für Qualitätssicherung und Wirtschaftlichkeit im Gesundheitswesen (IQWiG), Köln, Germany

## Abstract

**Background:**

The ankle brachial index (ABI) is an efficient tool for objectively documenting the presence of lower extremity peripheral arterial disease (PAD). However, different methods exist for ABI calculation, which might result in varying PAD prevalence estimates. To address this question, we compared five different methods of ABI calculation using Doppler ultrasound in 6,880 consecutive, unselected primary care patients ≥65 years in the observational getABI study.

**Methods:**

In all calculations, the average systolic pressure of the right and left brachial artery was used as the denominator (however, in case of discrepancies of ≥10 mmHg, the higher reading was used). As nominators, the following pressures were used: the highest arterial ankle pressure of each leg (method #1), the lowest pressure (#2), only the systolic pressure of the tibial posterior artery (#3), only the systolic pressure of the tibial anterior artery (#4), and the systolic pressure of the tibial posterior artery after exercise (#5). An ABI < 0.9 was regarded as evidence of PAD.

**Results:**

The estimated prevalence of PAD was lowest using method #1 (18.0%) and highest using method #2 (34.5%), while the differences in methods #3–#5 were less pronounced. Method #1 resulted in the most accurate estimation of PAD prevalence in the general population. Using the different approaches, the odds ratio for the association of PAD and cardiovascular (CV) events varied between 1.7 and 2.2.

**Conclusion:**

The data demonstrate that different methods for ABI determination clearly affect the estimation of PAD prevalence, but not substantially the strength of the associations between PAD and CV events. Nonetheless, to achieve improved comparability among different studies, one mode of calculation should be universally applied, preferentially method #1.

## Background

The prevalence of peripheral arterial disease (PAD) is rising since life expectancy is steadily increasing. Intermittent claudication (IC) as a classical manifestation of PAD becomes evident in only a fraction of the affected patients, thus demonstrating that the course is predominantly asymptomatic [1,2]. The clinical importance of the early identification of PAD as a manifestation of generalised atherothrombotic disease has been increasingly acknowledged in the recent years: although limb loss is a rare event in patients with intermittent claudication [3], the presence of PAD is a powerful predictor of future cardiovascular and cerebrovascular events and of increased mortality [4-7]. In primary health care, history taking and physical examination are still the major tools for the diagnosis of PAD, which leads to substantial under-diagnosis of the disease [8,9]. Further, the positive predictive value of intermittent claudication or diminished peripheral pulses seems not be sufficiently high [10,11].

The ankle brachial index (ABI) offers a simple and effective method of objectively documenting the functional state of the circulation in the lower limb and thus for the diagnosis of lower extremity PAD. The measurement of ABI can be performed in general practice using inexpensive equipment and is an efficient tool which improves the quality and efficiency of primary care with regard to PAD [12,13]. A normal ABI, indicating good blood flow to the extremity, is around 1.1 [14]. However, in a patient with compromised perfusion of the lower limb, the index is much lower than this. In this case, an ABI below 0.9 is routinely found, and an index below 0.4 indicates severe ischaemic symptoms [15]. Compared to angiography, an ABI less than 0.9 is 90% sensitive and 98% specific for a stenosis of 50% or more in leg arteries [13,16] and, among well-trained operators, the test-retest reliability is excellent [12, 17].

Assessment of ABI is performed by dividing the ankle systolic pressure by the highest brachial systolic pressure [15]. While this procedure allows various possibilities for calculating the ABI, only one mode of calculation is recommended by experts [18]. It appears predictable that different modes of ABI calculation result in different PAD prevalence estimates. Up to now, however, how the different modes of ABI calculation affect the PAD prevalence estimates and the association of PAD with other concurrent manifestations of atherothrombotic disease have not been systematically investigated.

## Methods

### Ethics

The protocol of the German Epidemiological Trial on Ankle Brachial Index (getABI) was approved by the Ethics Committee of the University of Heidelberg in 2001. All participating patients gave written informed consent. The study was conducted according to the 'Good Epidemiological Practice' recommendations issued by the 'German Working Group Epidemiology' [19].

### Study design

The (getABI) study is a large-scale epidemiological study with a cross-sectional and longitudinal part. Results reported in this paper refer to the cross-sectional part only. The methods and design of the study have been described elsewhere in greater detail [20,21]. Briefly, the central study co-ordinating centre selected 34 vascular physicians on the basis of their expertise in PAD. These vascular physicians, serving as centres of excellence, were evenly distributed geographically nation-wide, and each suggested on average 10 general practitioners to the central co-ordinating centre. Appropriate statistical methods were used to check that the distribution of the 344 GPs was representative in terms of location (post codes) and education (internists serving as GPs, and general physicians) for the total number of approximately 56,000 primary care physicians in Germany. Several weeks before the start of the study, in 34 regional meetings the centres of excellence instructed the GPs and their support staff about the requirements of the study and trained them in the clinical measurements, focusing particularly on ABI assessment.

### Patients

A prevalence assessment of primary care attendees, irrespective of their reason for seeing the doctor, was conducted within a pre-specified week in October 2001. In each practice, the gender and age category of all patients attending the practice and seeing the doctor were recorded in a log-file for each day of the week. The only exclusion criterion was life expectancy < 6 months. A total of 20 (in exceptional cases up to 25) eligible patients fulfilling the inclusion criteria (age ≥65 years, patient being legally competent and able to co-operate appropriately and providing written informed consent) were recruited, preferably as evenly as possible over this week in order to avoid selection bias. The data management centre was notified by fax about the inclusion of the patients on a daily basis. The baseline visit with the initial study examinations as specified below was to be performed within 6 weeks after the recruitment week. Further investigations included patient and medication history, physical examination and central laboratory tests at entry. The medical history as assessed at baseline included the following conditions: cardiovascular diseases (i.e. myocardial infarction, angina pectoris, revascularisation procedures), cerebrovascular diseases (i.e. stroke, transitory ischaemic attacks, or revascularisation procedures on the carotids), peripheral PAD (i.e. a history with regard to gangrene or amputation [minor and major form] of the lower extremities on account of PAD, IC [pain in the calf muscles while walking or during other exertion, which disappears within 10 min at rest], or revascularisation procedures on the peripheral arteries). In addition, the patients completed the WHO Rose questionnaire on intermittent claudication [22].

#### Determination of ankle brachial index

During 34 regional study workshops the GPs were specifically trained by certified specialists in vascular medicine (centres of excellence) to perform ABI measurements under standardised conditions [20], using a Doppler ultrasonic device (Kranzbühler 8 MHz, Solingen, Germany). The blood pressure cuff was used to measure systolic blood pressure in the brachial artery in both arms by use of the Doppler detector in the antecubital fossa. It was then applied to the distal calf, and the Doppler probe was used to determine systolic blood pressure at the left and right posterior and anterior tibial arteries after a 5-minute rest. Measurements were performed in supine position with the upper body as flat as possible, since measurements in the sitting or semi-sitting position may result in a substantial increase in tibial artery blood pressure. Calculation of the ABI was based on the following methods (see also Table 1): the highest arterial pressure of each leg (either posterior tibial or anterior tibial artery above the ankle; method #1), the lowest pressure (either posterior tibial or anterior tibial artery above the ankle; #2), only the systolic pressure of the tibial anterior artery (#3), and only the systolic pressure of the tibial posterior artery (#4). Furthermore, the ABI was also determined after exercise, however, only in the tibial posterior artery of each leg (#5). The ABI was calculated as follows: the ABI for each leg equals the ratio of the respective ankle pressure as determined by methods #1–#5 to the average of the right and left brachial artery pressures, unless there is a discrepancy of 10 mmHg in blood pressure values between the two arms. In such a case, the higher reading was used for the ABI. For the further analyses, the higher of the two ABI values obtained from the left and the right ankle was used.

**Table 1:** Description of the methods used for determination of the ankle-brachial index (ABI)

**Method of ABI determination**	**Procedure (nominator in the ABI calculation)**
#1	highest systolic pressure (tibial anterior/posterior artery)
#2	lowest systolic pressure (tibial anterior/posterior artery)
#3	only systolic pressure of tibial anterior artery
#4	only systolic pressure of tibial posterior artery
#5	after exercise (tibial posterior artery only)

The blood pressure measurements were performed on each leg as described in the methods section. The denominator in all calculations was the average of the right and left brachial arterial pressure (unless discrepancy of ≥10 mmHg, which led to the use of the higher pressure).

### Determination of PAD

An ABI < 0.90 in either leg was considered as evidence of PAD for all five determination methods. However, the classification of patients according to the ABI was modified as follows: patients with an ABI > 1.5 and no history of peripheral revascularisation and/or amputation on account of PAD were excluded from the analysis. An ABI of 1.5 or higher is consistent with poorly compressible leg arteries and is unreliable for gauging arterial perfusion accurately. Additionally, patients with a history of peripheral vascular revascularisation and/or limb amputation and ABI values ≥0.9 were classified as PAD patients.

### Statistical analysis

For the association between PAD status (determined using the different modes of ABI calculation and using a cut off value of 0.9) and the presence of cardiovascular events (in the patient's history), odds ratios and sensitivities for the 'detection' of the history of cardiovascular events (with corresponding specificities) were calculated. In addition, the association between ABI (determined using the different modes of calculation) and prevalent cardiovascular events was described by receiver operating characteristic (ROC) curves. Statistical analyses were performed with SAS version 8.2 (SAS Institute Corp., Cary, NC, 1999).

## Results

### Description of the sample

A total of 27,486 patients aged 65 years or older (10,722 men and 16,764 women) were screened by their GP in the recruitment week of the study. Only patients with written informed consent could be included, and this was provided for this long-term study by only about one quarter of patients (n= 6880). The age distribution of the patients was consistent with the age distribution in Germany [23] (Table 2). Compared to the patients screened, the percentage of recruited patients aged between 65 and 74 was slightly higher, whereas that of patients aged ≥80 years was somewhat lower. Thus, compared to the general population, the younger patients were over-represented in the study, whilst the older were slightly under-represented (Table 2). The gender distribution of included patients was very similar to that of the general population in Germany (not shown).

**Table 2:** Age distribution of screened and included patients compared with the age distribution in Germany within the respective age categories

Age category (years)	Age distribution in Germany (%)	Patients screened for getABI (%)	Patients included in getABI (%)
65–69	30.1	30.9	34.6
70–74	26.6	26.5	32.1
75–79	21.4	20.0	21.7
80–84	9.7	13.3	9.4
≥85	12.2	9.3	2.2

Source for age distribution in Germany: [23].

### Prevalence estimates of PAD

The percentage of patients with symptomatic PAD was 9.2%, which included intermittent claudication (4.6%), and/or positive Rose questionnaire (2.3%), and/or peripheral event (2.3%).

**Figure 1:**
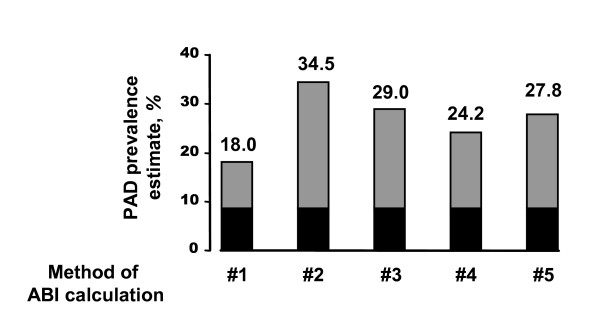
Prevalence estimates for PAD using different methods for ABI calculation. PAD was defined by an ABI value < 0.9 *(grey bars) *or clinical evidence of PAD *(black bars)*. Clinical evidence of PAD included positive Rose questionnaire, intermittent claudication and peripheral vascular event.

As shown in Fig. 1, the PAD prevalence estimate was profoundly affected by the mode of ABI calculation. While method #1 yielded the lowest prevalence estimate (18.0%), method #2 resulted in a value nearly double that (34.5%). The differences between methods #3, #4 and #5 were less pronounced (29.0%, 24.2% and 27.8% respectively).

### Association between PAD and cardiac events

**Figure 2:**
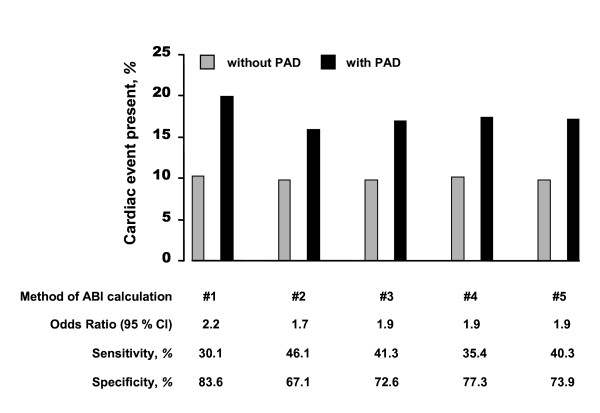
Association between PAD and history of cardiac event using odds ratios (OR). PAD was defined by an ABI value < 0.9 or clinical evidence of PAD, while history of cardiac event was evident after myocardial infarction or coronary revascularisation. OR, sensitivity and specificity are shown for the different modes of ABI calculation.  Sensitivity and specificity are given for the 'detection' or 'exclusion', resp., of a history of cardiac events.

The association between PAD and cardiac events was influenced by the mode of ABI determination when odds ratios (OR) were used for the description of this association. Cardiac events were classified as myocardial infarction or coronary revascularisation (*n *= 821 patients). As demonstrated in Fig. 2, the univariate OR at the cut-off point of 0.9 was highest in method #1 (2.2) and lowest in method #2 (1.7). In methods #3–#5, the OR was 1.9 and did not differ between the modes of ABI calculation. The sensitivity for detection of a history of cardiac events was highest in method #2 (46.1%) and lowest in method #3 (30.1%). In contrast, the specificity was highest in method #1 (83.6%) and lowest in method #2 (67.1%). In methods #3–#5, the respective sensitivities and specificities were in the same range and did not differ appreciably between the ABI calculation methods.

**Figure 3:**
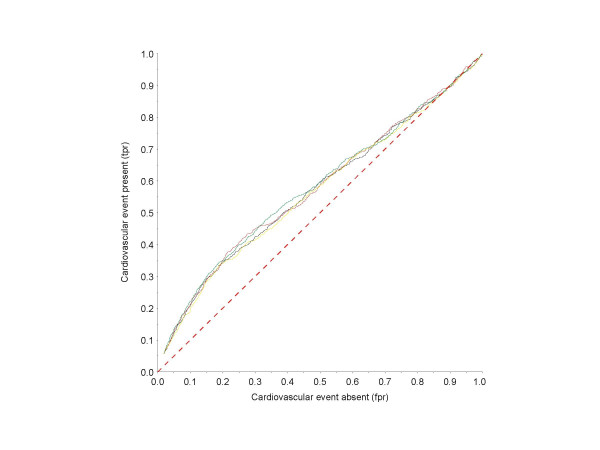
ROC curves for the association between ABI values (according to different methods for ABI calculation) and the history of cardiovascular events (myocardial infarction or coronary revascularisation). *Black line*, method #1; *red line*, method #2; *yellow line*, method #3; *green line*, method #4. The red dashed line represents the line of identity of tpr and fpr.

However, using receiver operating characteristic (ROC) methodology, the association between PAD according to the different modes of ABI calculation and cardiac events was nearly the same, with only slight differences between the respective ROC curves (Fig. 3).

## Discussion

Determination of ABI allows the non-invasive and reliable detection and quantification of PAD. It is one of the most widely used methods in the epidemiology of PAD since it can be easily performed in large-scale studies, with low variability of measurements between different observers [12,17,24]. At the same time, it is increasingly used as screening measure in primary care. However, different modes of ABI calculation are used in the literature. In some reports, the highest arterial pressure in each leg is used, whilst in others the lowest arterial pressure serves to determine the ABI. Alternatively, only the tibialis posterior or dorsalis pedis pressure is used, or the pressures of one leg are averaged [18,24-27]. However, the optimal method for ABI calculation for estimating the prevalence of PAD and predicting mortality and other outcomes in PAD has to our knowledge not been determined.

This is the first large-scale epidemiological study to screen an unselected sample of patients in primary care for the prevalence of PAD using different methods for the calculation of the ABI. The data confirm the high prevalence of PAD in primary care [28,29], indicating that – at least – about every fifth subject aged ≥65 years, is a PAD patient.

In our study, and as recommended by the American Heart Association [30], taking the higher of the two systolic pressures (method #1, tibial posterior and anterior artery) resulted in the most conservative estimate of the prevalence of PAD. However, at ABI values > 1.5, the presence of poorly compressible ankle arteries due to calcified vessels should be taken into account. Incompressible arteries are frequently observed in diabetics and haemodialysis patients and may result in incorrectly high ABI values [31].

Modes of ABI calculations in which the dorsalis pedis (which is the distal extension of the anterior tibial artery) pressure (method #3) serves as the numerator may be misleading and not generally suitable in primary care, since an absent dorsalis pedis signal due to hypoplasia is described in 8–12% of healthy subjects without PAD [11,32]. The smaller diameter of the dorsalis pedis artery relative to the posterior tibial artery may be more difficult for general physicians to locate with the Doppler probe (leading to an underestimate of the true pressure), and in addition, artery pressures due to pulse wave reflection in smaller vessels may be different in the two vessels. [14]

Thus, if the numerator were defined by the dorsalis pedis pressure (method #3) or the lowest ankle pressure (method #2) (which would be 0 if the dorsalis pedis pressure is not detectable), the value 0 would be attributed to these legs although PAD would be absent. Likewise, in a smaller study in healthy subjects, Aboyans et al. [26] disqualified methods involving the lower arterial pressure at the ankle, since this method cannot distinguish between hypoplastic or obstructed arteries. As in our approach, that study also recommended the use of the higher arterial pressure at the ankle for calculation of the ABI, which yielded a PAD prevalence estimate of about 5% at the cut-off point of 0.9. This lower value can be explained by the fact that patients with history of PAD were excluded in the study by Aboyans et al. Moreover, the cohort was significantly younger, with only 26% of the patients being > 65 years [26].

In another approach, McDermott et al. correlated three modes of ABI determination to leg functioning parameters in PAD patients [33]. As in the present observations, using the lower of the two arterial pressures at the ankle (method #2) resulted in a significantly higher PAD prevalence. However, their results suggested that the ABI, determined by averaging the dorsalis pedis and posterior tibial arterial pressures in each leg, may be most predictive of walking endurance and walking speed in patients with peripheral arterial disease. In contrast to that study, which was performed with regard to leg functioning parameters on selected patients with high prevalence of PAD, the present investigation was performed on a large-scale general population aged 65 years or older. Consequently, the results may not be directly comparable.

Another finding is of considerable importance for the practical use of the ABI for screening purposes. While the different modes of ABI calculation profoundly affected the estimation of PAD prevalence, there was no major effect on the association between other concomitant manifestations of atherothrombotic disease, i.e. cardiovascular events. ROC analyses were performed to describe the relationship between sensitivity and specificity of different ABI values (according to different modes of calculation) with respect to history of cardiovascular events. Interestingly, the respective ROC curves differed only marginally between the diverse modes of calculation, indicating that the strength of association between the ABI and cardiovascular events is largely independent of the mode of ABI determination. Thus, an abnormal ABI, irrespective of the mode of calculation, is associated with the same prevalence of cardiovascular events at a "mode-specific" cut-off point.

For the assessment of exercise ABI, in contrast to a previous methodological study [14] that used the posterior tibial artery *or *the dorsalis pedis artery (which is the distal extension of the anterior tibial artery), due to the reasons given above [14,32] we chose to focus on measurements on one vessel, the posterior tibial artery. Of note, even the determination of the ABI after exercise did not increase the strength of association with concurrent (or history of) cardiovascular events. Since ABI calculation after exercise is a demanding procedure in terms of time and manpower, and since there is no obvious advantage over other methods, this approach may not be suitable for routine determination of the ABI in general practice.

## Conclusion

To summarise, for improved comparability of data in epidemiological and clinical studies employing the ABI, one mode of ABI calculation should be universally used. As previously suggested by the American Heart Association, taking the higher of the two arterial pressures at the ankle may be the most suitable procedure for ABI determination in primary care.

## Competing interests

MM is an employee of Sanofi-Aventis, Germany, the manufacturer of clopidogrel which is used, among others, in the indication peripheral arterial disease. The other authors declare that they have no competing interests.

## Authors' contributions

CD and HJT are on the steering committee of the getABI study; all other authors - with the exception of SL - are on the advisory board. SL had substantial impact on the design of the getABI study. JRA, RLH, GT and MM participated in the study design and   interpreted the results. SL, CD, HJT and DP planned or performed the statistical work required for this paper. DP and SL wrote the paper. All authors read and approved the final manuscript.

## Pre-publication history

The pre-publication history for this paper can be accessed here:


